# Association of thyroid function with arterial pressure in normotensive and hypertensive euthyroid individuals: A cross-sectional study

**DOI:** 10.1186/1756-6614-1-3

**Published:** 2008-09-29

**Authors:** Katerina Saltiki, Paraskevi Voidonikola, Kimon Stamatelopoulos, Emily Mantzou, Christos Papamichael, Maria Alevizaki

**Affiliations:** 1 Endocrine Unit, Evgenidion Hospital, Athens University School of Medicine, Athens, Greece; 2 Endocrine Unit, Dept Medical Therapeutics, Alexandra Hospital, Athens University School of Medicine, Athens, Greece; 3 Vascular Laboratory, Dept of Medical Therapeutics, Alexandra Hospital, Athens University School of Medicine, Athens, Greece

## Abstract

**Background:**

Overt hypothyroidism has been associated with arterial hypertension and increased arterial stiffness. Results in euthyroid individuals have been conflicting. We investigated associations of thyroid function with systolic (SAP) and diastolic (DAP) arterial pressure in euthyroid subjects.

**Methods:**

311 euthyroid individuals (185 women, mean age 43.9 ± 9) without a history of diabetes attending a preventive medicine program were examined. Subjects receiving thyroxine (10.6%) were excluded; 19.3% had hypertension, 43% had a family history for hypertension. TSH, fT4, thyroid autoantibodies, insulin, glucose were measured. The "fT4.TSH product", which has been suggested as a T4 resistance-index, was calculated.

**Results:**

TSH range was 0.1–8, median 1.4 mU/L, fT4 range was 11.5–25.2 pmol/L, median 17.4. TSH and the "fT4.TSH product" were positively associated with DAP (p < 0.03, for both associations). In the subgroup of individuals with TSH levels 0.36–2.5 mU/L, both TSH and the "fT4.TSH product" were positively correlated with SAP (r = +0.133 p = 0.044, r = +0.152 p = 0.026) and DAP (r = +0.243 p < 0.001, r = +0.252 p < 0.001 respectively); in multivariate analysis the "fT4.TSH product" was a significant predictor of DAP independently of HOMA-IR and BMI (p < 0.001). Similar associations were found when only the non-hypertensive subjects were analysed (p = 0.004). Hypertensive patients had higher TSH levels (p = 0.02) and belonged more frequently to the subgroup with TSH > 2 mU/L (35.3% vs 21.3%, p = 0.045).

**Conclusion:**

In euthyroid individuals the association of thyroid function with diastolic arterial pressure remains significant even when a stricter "normal range" for TSH levels is considered. The "freeT4.TSH" product appears to be an even stronger predictor of DAP, independently of HOMA insulin resistance index and obesity.

## Background

Thyroid hormones influence cardiovascular function [1,2] and modulate the vascular response [3]. Overt hypothyroidism may be associated with hypertension and various adverse cardiovascular effects [4-9].

Subclinical hypothyroidism has also been associated with arterial hypertension, mostly diastolic [10-12], as well as with atherosclerosis [13,14] and coronary heart disease [15,16] in both sexes. However these findings have not been consistent. In overt and especially in subclinical hypothyroidism normotensive patients are quite frequently observed particularly in younger age groups [17-20]. Thus the risk of mild thyroid failure for cardiovascular disease and, subsequently, the need for a therapeutic intervention in this group, remain controversial [1,19,21].

It is interesting that such associations have also been reported for euthyroid individuals. TSH variation within the normal range has been associated with alterations in various cardiovascular parameters including arterial pressure [11,22,23] and lipid profile [11,24]. Early markers of atherosclerosis such as increased intima media thickness [25] and impaired endothelial function [26-28] may be detected among patients with borderline thyroid function. It has further been reported that hypertensive patients may have a tendency for impaired thyroid function [29]; however these results have not always been confirmed [20].

The mechanism through which slightly impaired thyroid function might influence arterial blood pressure is not clear; increased systemic vascular resistance, premature atherosclerosis and increased arterial stiffness may all be involved [2,30]. It has been argued that in the hypothyroid state, the sympathetic and the renin-angiotensin-aldosterone systems are possibly implicated in the homeostasis of arterial pressure [31,32]. Thyroxine replacement therapy may reverse the cardiovascular effects and may result in the regulation of arterial pressure in subjects with subclinical hypothyroidism [8,32-35]. However, it has been suggested that the mechanisms involved in these associations may not be attributed to impaired thyroid hormone action; genetic factors alone may influence the regulation of both arterial blood pressure and circulating TSH levels as well as the individually determined set point of thyroid function [28].

The purpose of our study was to investigate associations of thyroid function parameters with systolic (SAP) and diastolic (DAP) arterial pressure in clinically euthyroid subjects.

## Methods

We studied 311 euthyroid individuals (185 women, 126 men, mean age 43.9 ± 9) who visited the cardiovascular laboratory in response to an announcement for free examination for unrecognized features of the metabolic syndrome in the outpatients' clinic of our hospital over a period of 12 months. Thyroid function tests were included in the protocol.

Exclusion criteria were a history of diabetes mellitus (previously diagnosed according to the American Diabetes Association criteria), overt coronary heart disease or a previous history of stroke. The presence of hypertension was defined as systolic and/or diastolic blood pressure higher than 139 mmHg and/or 89 mmHg, respectively and/or current use of antihypertensive drugs.

Patients receiving thyroxine as well as those who were on drugs possibly affecting thyroid function such as lithium, amiodarone or γ-interferon were excluded from the analysis (10.6%). Of the studied individuals included in the analysis (165 women, 113 men) 19.3% had hypertension and 43% had a family history for hypertension. The study was approved by the institutional Ethics Committee and all subjects gave their informed consent.

Using a standard mercury sphygmomanometer and after 5 minutes of rest, three consecutive measurements of arterial pressure were obtained while all subjects were in a sitting position and the mean was calculated. Height and weight were also measured with subjects wearing indoor clothes without shoes. Body mass index (BMI) was calculated according to the formula weight (kg)/height (m^2^). Waist and hip perimeter (cm) measurements were performed and waist to hip ratio (W/H R) was used to evaluate fat distribution. Current drug therapy and clinical history were also recorded. Ninety one (55.1%) of the women participating in the study were premenopausal.

Fasting blood samples were obtained by venipuncture between 08:00–09:00 h. Serum TSH and free thyroxine were measured using chemiluminescent immunometric assays with the DPC Immulite 200 (Siemens). Serum anti-thyroid antibodies (antiTPO, antiTG) analysis was performed by RIA using the reagents Brahms DINOtest (Brahms diagnostica GmbH, Berlin). Reference range was: TSH 0.36–4 mU/L, fT4 9–25 pmol/L, antiTPO < 60 IU/ml, antiTG < 60 IU/ml. Data were also analysed using a TSH cutoff of either 2 or 2.5 mU/L. Glucose levels (measured immediately using an automated analyser Integra 400, Roche) and insulin levels (in specimens kept frozen at -20°C until analysis was performed by IRMA, Biosource Europe SA, Nivelles, Belgium) were also estimated. Basal insulin resistance index (Homeostasis Model Assessment, HOMA) was calculated according to the formula: Insulin resistance = FI × G/22.5, where FI = fasting insulin (μU/ml) and G = fasting glucose (mmol/L).

The "fT4.TSH product", derived by multiplying TSH by fT4, was calculated. Previous studies have suggested that this index, referred to as T4 resistance-index, may determine the fT4-TSH set-point for each person. TSH appears to contribute by 85% to the fT4.TSH variance [28,36].

### Statistical analysis

Statistical analysis was done using the SPSS statistical package. All descriptive data are presented as mean ± SD. Linear regression analysis was used to investigate correlations between continuous variables. In the multivariate analysis we included as possible confounders all the variables for which there was some correlation which was statistically significant or which showed a tendency to be significant in the univariate analysis. Student's t – test was used to compare mean values between groups where the distribution was normal. Chi – square test with Yates' continuity correction was used, or chi – square for linear association as appropriate.

## Results

TSH range was 0.1–8, median 1.4 mU/L; fT4 range was 11.5–25.2, median 17.4 pmol/L. The clinical and biochemical characteristics of the population are shown in table 1.

**Table 1:** Baseline characteristics of the studied population

	%	Mean ± SD	Range
Sex (men)	40.5		
Age (years)		43.9 ± 9	34.9–52.9
BMI		26.9 ± 4.7	19.0–43.3
Waist-to-hip Ratio		0.87 ± 0.12	0.52–1.39
Hypertension	19.3		
Dyslipidemia	41		
Family history of hypertension	43		
Smoking	42		
TSH (mU/L)		1.6 ± 1.01	0.1–8.0
fT4 (pmol/L)		17.8 ± 2.2	11.5–25.2
fT4.TSH product		26.4 ± 15.8	1.04–92
Systolic blood pressure (mmHg)		119.7 ± 20.3	85–195
Diastolic blood pressure (mmHg)		77.2 ± 12.3	55–120
HOMA-IR index		1.88 ± 1.4	0.4–11.0

**Figure 1:**
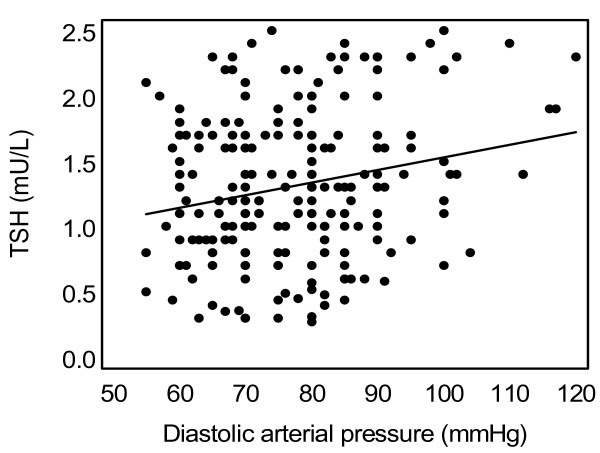
Association of diastolic arterial blood pressure with TSH levels in the subgroup of apparently healthy subjects with TSH levels in the narrow "normal range" (0.36–2.5 mU/L) (r = +0.243, p < 0.001).

**Figure 2:**
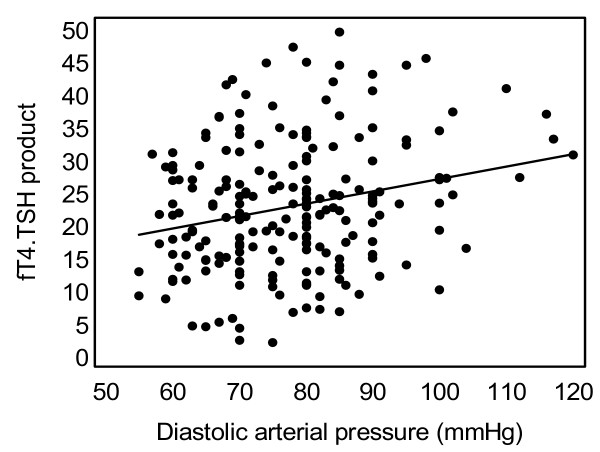
Association of diastolic arterial blood pressure with the "fT4.TSH product" in the subgroup of apparently healthy subjects with TSH in the narrow "normal range" (0.36–2.5 mU/L) (r = +0.252, p < 0.001).

TSH and the "fT4.TSH product" were positively associated with diastolic arterial pressure (DAP) (r = +0.134, p = 0.029 and r = +0.156, p = 0.014 respectively). In the subgroup of individuals with TSH levels 0.36–2.5 mU/L (n = 238), TSH levels were positively correlated with systolic (r = +0.133, p = 0.04) and diastolic arterial pressure (r = +0.243, p < 0.001, figure 1). Similarly, in the same subgroup, the "fT4.TSH product" was positively correlated with SAP (r = +0.152, p = 0.026) and DAP (r = +0.252, p < 0.001, figure 2) respectively. A multivariate analysis (step model) was performed considering associations of DAP with age, sex, cardiovascular risk factors such as HOMA-IR and BMI and thyroid function parameters. In this model, the "fT4.TSH product" was a significant independent predictor of DAP (table 2). The association of DAP with TSH was no longer significant when age, BMI and HOMA-IR were taken into account. Similarly, when only the normotensive subjects with TSH levels 0.36–2.5 mU/L were analysed (n = 203), TSH levels and the "fT4.TSH product" showed positive association with DAP (r = +0.208, p = 0.004 and r = +0.212, p = 0.004 respectively, Pearson's correlation).

**Table 2:** Stepwise multiple regression model for predicting diastolic arterial pressure in apparently healthy individuals with TSH 0.36–2.5 mU/L

Variable	Predictor	Beta	T	Significance *P*	Overall R^2 ^(%)
Diastolic arterial pressure	Age	0.193	3.103	0.002	0.295 *P *< 0.001
	Sex	-0.222	-3.625	<0.001	
	HOMA-IR	0.203	2.705	0.007	
	BMI	0.167	2.154	0.032	
	"fT4.TSH product"	0.210	3.515	0.001	

Hypertensive patients had higher TSH levels (1.92 ± 1.04 vs 1.54 ± 1.3, p = 0.02, t-test) and belonged more frequently to the subgroup with TSH > 2 mU/L compared to normotensive ones (35.3% vs 21.3%, p = 0.045 x^2^-test). Normotensive subjects with a family history for hypertension belonged more frequently to the subgroup with TSH > 2 mU/L although this finding was not significant (p = 0.056, Pearson chi-square).

There were no significant differences in the occurrence of hypertension in the patients with positive thyroid auto-antibodies (either antiTG or antiTPO).

In the subgroup with subclinical hyperthyroidism (n = 17, 5.7%) there were no associations between TSH levels and systolic and/or diastolic blood pressure.

## Discussion

Our study has demonstrated that in euthyroid individuals with TSH levels in the narrow "normal range" (0.36–2.5 mU/L), diastolic as well as systolic arterial blood pressure is positively associated with TSH levels. These associations remained significant even when only normotensive subjects were considered. Similar positive associations have also been reported by other investigators [11,12]. Asvold et al [22] in a large population study which included more than 30000 euthyroid subjects, found a positive linear association between systolic and diastolic arterial pressure and TSH levels. Furthermore, the fifth Tromso study showed that systolic and diastolic blood pressure were higher in euthyroid individuals belonging to the highest versus the lowest serum TSH quartile of the normal range [23]. On the other hand, in another recent community-based study mean DAP and SAP did not differ between individuals with subclinical hypothyroidism and euthyroid subjects [20]. Similarly, no such associations were reported in other studies concerning either younger [34,37] or older [14,17] individuals; however in these studies there were significant associations of mild thyroid failure with other cardiovascular parameters such as left ventricular diastolic dysfunction [34] or atherosclerosis and coronary heart disease [14,37].

We have further demonstrated that the "fT4.TSH product" is also positively associated with diastolic as well as with systolic blood pressure. This association was independent of age, sex, insulin resistance and obesity. The same result was found even when only normotensive subjects were considered for the analysis. This marker has been rarely used in the literature and may reflect an individually determined fT4-TSH relationship [28,36]; it may not represent thyroid function but it may be indicative of an altered sensitivity of peripheral tissues to thyroid hormones. This means that higher TSH levels for a standard value of freeT4 are associated with higher arterial blood pressure and thus probably with impaired peripheral vascular function [28]. There are no reports in the literature for similar associations concerning arterial pressure. Although the significance of this product cannot be clearly defined, it is interesting that recently vascular indices have again been associated with this marker. Fernandez-Real et al reported significant associations of the same marker with insulin sensitivity and endothelial function in euthyroid individuals [28]. In this study, patients with reduced endothelium-dependent vasodilatation had "fT4.TSH product" values above the median and also increased TSH levels. Based on these results a hypothesis of possible genetic variations which could influence vascular function and serum TSH as well as the individually determined thyroid set point, might be supported [28,38].

Another finding in our study concerns the subjects with a history of hypertension. Higher TSH levels were associated with a history of hypertension. Moreover, hypertensive subjects belonged more frequently to the subgroup with TSH > 2 mU/L. As we have previously shown, TSH values above these levels, may be associated with endothelial dysfunction [26] and increased arterial stiffness [30] compared to euthyroid subjects with TSH levels lower than 2 mU/L. Similar results have been reported in the population study of Asvold et al [22]. They showed that when subjects with TSH levels in the upper part of the normal range (3–3.5 mU/L) were compared to those with TSH levels in the lower part of the normal range (0.5–0.99 mU/L), the odds ratio for hypertension in the former group was 1.98 for men and 1.23 for women, respectively. In another study, by Iqbal et al, TSH levels were higher among individuals with diastolic hypertension [23]. Finally, Gumieniac et al showed that euthyroid hypertensive subjects had slightly impaired thyroid function with lower fT4 index and higher TSH levels compared to normotensive subjects [29]. On the other hand Walsh et al reported no differences in the prevalence of hypertension in subjects with subclinical hypothyroidism compared to euthyroid ones, after adjustment for age and sex [20]. In another large study concerning an older population, no differences were observed in the prevalence of hypertension, cardiovascular outcomes and mortality between the overt hypothyroidism, subclinical hypothyroidism and normal thyroid function groups [19]. In agreement with other studies we did not find any associations between thyroid autoantibody positivity and the presence of hypertension [20].

Finally, normotensive individuals with a family history of hypertension belonged more frequently to the subgroup with TSH > 2 mU/L although this association just missed statistical significance. A similar finding has been reported by Gumieniac et al in a study of hypertensive families [38]. In this study normotensive euthyroid subjects with a family history of hypertension had higher TSH levels compared to those with a negative history. In the same study, a familial aggregation of high-normal TSH levels was observed; these investigators speculated that there may be common genetic variants which affect both the pituitary-thyroid axis set point and the regulation of arterial pressure. As it has been mentioned previously, "the fT4.TSH product" could be a phenotypic marker of each individual's set point of thyroid function, probably genetically determined. Thus, one might suggest that our results indirectly concur with the observations reported by Fernandez-Real et al [28] and by Gumieniac et al [38]. Studies in spontaneously hypertensive rats have confirmed that arterial pressure regulation is in part mediated by TRH [39]; similarly, polymorphic variants of genes such as the TSH receptor gene or the type 2 iodothyronine deiodinase genes could be implicated in both insulin resistance and the regulation of arterial pressure and TSH levels [40,41].

We did not find associations between arterial pressure and TSH at the lower levels of the normal range or with the presence of subclinical hyperthyroidism. This could be due to the small sample of subjects with subclinical hyperthyroidism. In the literature there are reports for such associations [20,22] although these results are not consistent [42].

A limitation of our results was the relatively small number of studied individuals. However, several of our findings point to the same direction.

## Conclusion

In conclusion, in euthyroid individuals the associations of thyroid function with arterial blood pressure remain significant even when a stricter "normal range" for TSH levels is considered. The "freeT4.TSH" product appears to be an even stronger predictor of diastolic arterial blood pressure, independently of HOMA insulin resistance index and obesity.

## Abbreviations

BMI: Body Mass Index; DAP: Diastolic Arterial Pressure; fT4: free Thyroxine; HOMA-IR: Homeostasis Model Assessment-Insulin Resistance Index; SAP: Systolic Arterial Pressure; T3: Triiodothyronine; T4: Thyroxine; TSH: Thyroid Stimulating Hormone; W/H R: Waist-to-Hip Ratio.

## Competing interests

The authors declare that they have no competing interests.

## Authors' contributions

KS drafted the manuscript and performed the statistical analysis. PV participated in the design of the study and collected the data. KS performed the statistical analysis. EM carried out the immunoassays. CP conceived the study. MA performed the statistical analysis, conceived the study and participated in its design and coordination. All authors read and approved the final manuscript.
